# Rotigotine Transdermal Patch in Parkinson’s Disease: A Systematic Review and Meta-Analysis

**DOI:** 10.1371/journal.pone.0069738

**Published:** 2013-07-23

**Authors:** Chang-Qing Zhou, Shan-Shan Li, Zhong-Mei Chen, Feng-Qun Li, Peng Lei, Guo-Guang Peng

**Affiliations:** Department of Neurology, The First Affiliated Hospital of Chongqing Medical University, Chongqing, P. R. China; National Hospital of Utano, Japan

## Abstract

**Background and Methods:**

The efficacy and safety of rotigotine transdermal patch in Parkinson’s disease (PD) were studied in some clinical trials. We performed a systematic review and meta-analysis of randomized controlled trials to evaluate the efficacy, tolerability, and safety of rotigotine transdermal patch versus placebo in PD.

**Results:**

Six randomized controlled trials (1789 patients) were included in this meta-analysis. As compared with placebo, the use of rotigotine resulted in greater improvements in Unified Parkinson’s Disease Rating Scale activities of daily living score (weighted mean difference [WMD] –1.69, 95% confidence interval [CI] –2.18 to –1.19), motor score (WMD –3.86, 95% CI –4.86 to –2.86), and the activities of daily living and motor subtotal score (WMD –4.52, 95% CI –5.86 to –3.17). Rotigotine was associated with a significantly higher rate of withdrawals due to adverse events (relative risk [RR] 1.82, 95% CI 1.29–2.59), and higher rates of application site reactions (RR 2.92, 95% CI 2.29–3.72), vomiting (RR 5.18, 95% CI 2.25–11.93), and dyskinesia (RR 2.52, 95% CI 1.47–4.32) compared with placebo. No differences were found in the relative risks of headache, constipation, back pain, diarrhea, or serious adverse events.

**Conclusions:**

Our meta-analysis showed that the use of rotigotine can reduce the symptoms of PD. However, rotigotine was also associated with a higher incidence of adverse events, especially application site reactions, compared with placebo.

## Introduction

Parkinson’s disease (PD) is a chronic and progressive neurodegenerative disorder characterized by the symptoms of resting tremor, rigidity, bradykinesia, and postural instability. These symptoms are largely caused by the progressive loss of dopaminergic neurons in the substantia nigra compacta, which ultimately reduces dopaminergic input to the striatum and other brain regions [1]. PD is the second most prevalent neurodegenerative disease, and affects approximately 1.7 million Chinese individuals [2].

Although clinical and pathologic studies have failed to demonstrate the neuroprotective effects of levodopa [3], levodopa is considered the most effective drug for managing PD [4]. However, the initial therapeutic efficacy is often impacted within a few years by the development of motor complications (fluctuations, dyskinesias) [5]–[8] that are intractable to treatment. It is now thought that pulsatile stimulation of striatal dopamine receptors, caused by intermittent administration of levodopa and erratic gastrointestinal absorption, plays a key role in the development of these motor complications [9].

Rotigotine is a non-ergot dopamine agonist that is suitable for transdermal delivery via skin patches [10]. Once-daily administration of a rotigotine transdermal patch (referred to here as rotigotine) provides stable plasma concentrations of rotigotine over 24 hours [11]–[13] and is associated with high compliance under clinical practice conditions [14]. Non-oral routes of rotigotine delivery are particularly useful in patients scheduled for surgery or in those with dysphagia [15]. In the past decade, some clinical trials have been carried out to evaluate the efficacy and safety of rotigotine versus placebo, but one of these trials obtained inconclusive results [16]. Furthermore, crystal formation was noted in some rotigotine patches and these crystal-related changes may reduce its bioavailability and clinical efficacy [17]. The marketing authorization of rotigotine was suspended by the United States Food and Drug Administration in 2008 because of this issue, although rotigotine was reintroduced in the United States in 2012. To date, no meta-analyses of rotigotine have been performed to evaluate its efficacy in PD. Therefore, we pooled all the results of randomized controlled trials that were published up to July 2012, and performed a comprehensive meta-analysis to evaluate the efficacy, tolerability, and safety of rotigotine in PD.

## Methods

### Literature Search

We conducted systematic literature searches of PubMed, EMBASE, Cochrane Library, and Web of Knowledge up to July 2012 without language limitations. A search strategy was performed using the following Medical Subject Headings (MeSH) and keywords: “rotigotine”, “rotigotine transdermal patch”, and “transdermal rotigotine” in combination with “Parkinson’s disease”, “Parkinson’s”, and “PD”. We also manually searched the references cited in clinical trial reports or reviews to identify additional relevant clinical trials. To maximize data requisition, we also contacted the authors whose articles contained insufficient information, where necessary.

### Study Selection

Randomized controlled trials of rotigotine transdermal patch were included if they reported efficacy data in the form of Unified Parkinson’s Disease Rating Scale (UPDRS) scores (activities of daily living [ADL] score, motor score, and/or the ADL and motor subtotal score) and safety data in the form of adverse events. Additional endpoints included: (1) overall withdrawals and withdrawals due to adverse events; and (2) serious adverse events. In trials for which there was more than one publication involving the same population, the most recent report was selected for analysis and the earlier articles were reviewed for missing data, where applicable.

### Validity Assessment

Two investigators (CQZ and FQL) independently evaluated all of the included trials. Any disagreement was resolved by discussion with a third investigator (PL). The validated Jadad scale was used to assess the methodological quality of the included trials [18]. This scale assesses inherent controllers of bias with the following quality assessment criteria: use of and methods for generating randomization, use of and methods for double-blinding, and description of patient withdrawals/dropouts. One point was given for each satisfied criterion. The aggregate score was calculated for each included trial and ranged from 0 (weakest) to 5 (strongest); trials scoring <3 were deemed to have lower methodological quality.

### Data Extraction

Using a pre-designed data extraction form, two investigators (CQZ and FQL) collected data independently with differences resolved by a third investigator (PL). The following information was collected from each trial: first author’s surname, year of publication, details of study design, methodological quality (assessed using Jadad criteria), patient characteristics (including gender, age, ethnicity, inclusion criteria, and disease severity at baseline), sample size, treatment of early- or late-stage disease, dose of rotigotine, duration of treatment, changes in UPDRS scores (ADL score, motor score, and the ADL and motor subtotal score), overall withdrawals, withdrawals due to adverse events, and the incidence of adverse events.

### Statistical Analysis

We combined the results of each trial by using standard meta-analytic methods to estimate the overall efficacy, tolerability, and safety. We classified trials according to the randomized treatment comparison: rotigotine (plus levodopa) versus placebo (plus levodopa).

The mean changes in UPDRS scores from baseline were treated as continuous variables and the weighted mean differences (WMDs) were calculated. Withdrawals and adverse events were treated as dichotomous variables and reported as relative risks (RRs) with 95% confidence intervals (CIs). The overall effect was tested using z scores calculated by Fisher’s z transformation, with significance set at P<0.05. Statistical heterogeneity between trials was evaluated by the χ^2^ and I^2^ tests, with significance set at P<0.10. If heterogeneity existed, the random-effect model was used to combine the results; otherwise, the fixed-effect model was used. Heterogeneity was only reported where it was statistically significant. Subgroup and sensitivity analyses were also performed. All data analyses were carried out using Stata software version 12.0 (Stata Corp LP, College Station, TX, USA).

## Results

### Search Results and Study Characteristics

**Figure 1:**
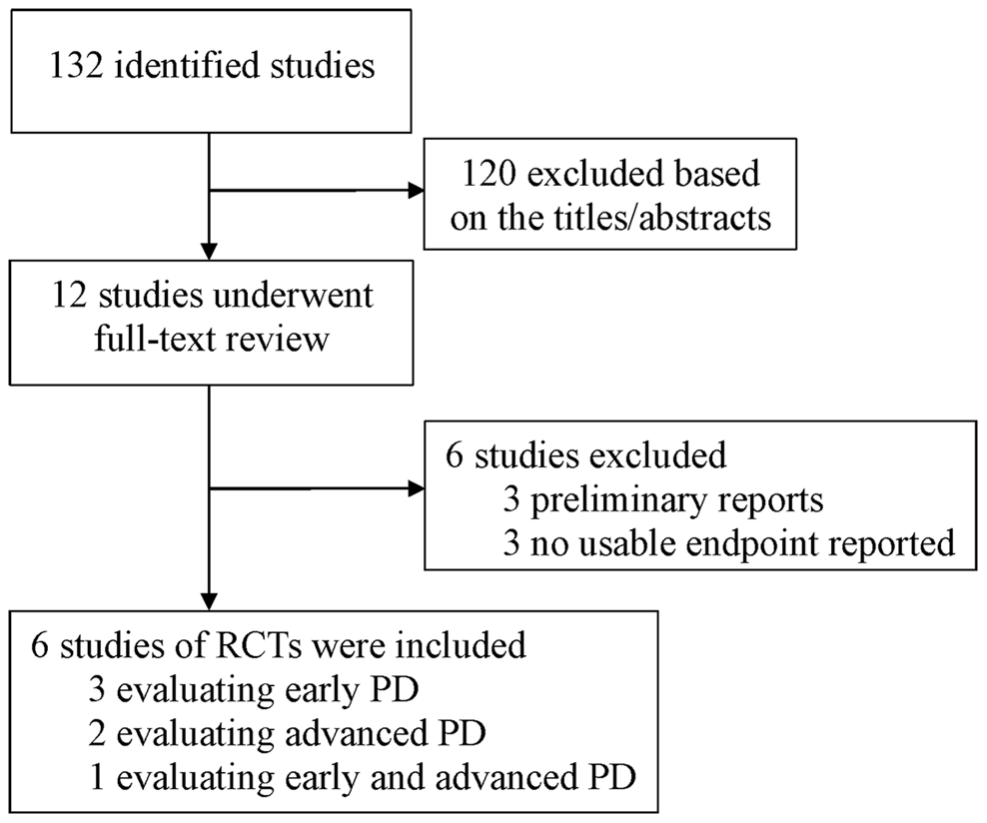
Flow diagram of literature search and selection process. Abbreviations: PD = Parkinson’s disease; RCT = Randomized controlled trial.

The literature searches identified 132 potential articles, of which 12 were randomized controlled clinical trials. Although the results of the study by Quinn et al. [16] were inconclusive, we did not obtain the full-text after contacting the author. Six large-scale randomized controlled trials involving 1789 patients were ultimately included in this meta-analysis. The study flow chart is presented in Figure 1. Of the studies included, three involved early PD patients who had not taken levodopa [19]–[21], two involved advanced PD patients who had already taken levodopa [22]–[23], and one involved a mixture of patients with the early or advanced PD [24]. Quality assessment demonstrated that all the trials had Jadad scores that ranged from 4 to 5. The main characteristics of these trials are summarized in Table 1.

**Table 1:** The main characteristics of the included randomized controlled trails.

Study, year	Study design (Jadad score)	Stage ofPD	Comparison^a^	Participants	Primary outcome data reported
PSG, 2003	MC DB Phase III RCT (5)	Early	Rotigotine vs Placebo (195/47),Rotigotine dose (mg/day): 2, 4,6, 8, Duration (w): 11	Mean age (y): 61/62, Men (%):67/49, Duration of PD (y):1.2/1.3	UPDRS ADL+Motor subtotal, dropouts, adverse events
Jankovic, 2007	MC DB Phase III RCT (4)	Early	Rotigotine vs Placebo (181/96),Rotigotine dose (mg/day):up to 6, Duration (w): 27	Mean age (y): 62/65, Men (%):68/60, Duration of PD (y):1.3/1.4	UPDRS ADL, Motor and subtotal, dropouts, adverse events
Giladi, 2007	MC DB Phase III RCT (5)	Early	Rotigotine vs Placebo (215/118),Rotigotine dose (mg/day):up to 8, Duration (w): 37	Mean age (y): 61/60, Men (%): 55/58, Duration of PD (y): 1.4/1.2	UPDRS ADL+Motor subtotal, dropouts, adverse events
LeWitt, 2007	MC DB Phase III RCT (5)	Advanced	Rotigotine+LD vs Placebo+LD(229/120), Rotigotine dose (mg/day):8, 12, Duration (w): 12	Mean age (y): 66/66, Men (%):65/62, Duration of PD (y):7.7/7.7	UPDRS ADL and Motor, dropouts, adverse events, “on” and “off” time
Poewe, 2007	MC DB Phase III RCT (5)	Advanced	Rotigotine+LD vs Placebo+LD(201/100), Rotigotine dose (mg/day):up to 16, Duration (w): 29	Mean age (y): 64/65, Men (%):66/71, Duration of PD (y):8.9/8.5	UPDRS ADL and Motor, dropouts, adverse events, “on” and “off” time
Trenkwalder, 2011	MC DB Phase III RCT (5)	Mixed	Rotigotine±LD vs Placebo±LD(191/96), Rotigotine dose(mg/day): up to 16, Duration(w): 23	Mean age (y): 65/64, Men (%):64/44, Duration of PD (y):4.6/4.9	UPDRS ADL and Motor, dropouts, adverse events

Abbreviations: MC = multicenter; DB = double-blinded; vs = versus; RCT = randomized controlled trial; Y = year; W =  week; UPDRS = unified Parkinson’s disease rating scale; LD = levodopa; ADL = activities of daily living; PSG = Parkinson study group.

a ±LD indicates that trial design allowed levodopa to be added to the randomized treatment; the dose of rotigotine is expressed as the delivered dose.

### Efficacy

**Figure 2:**
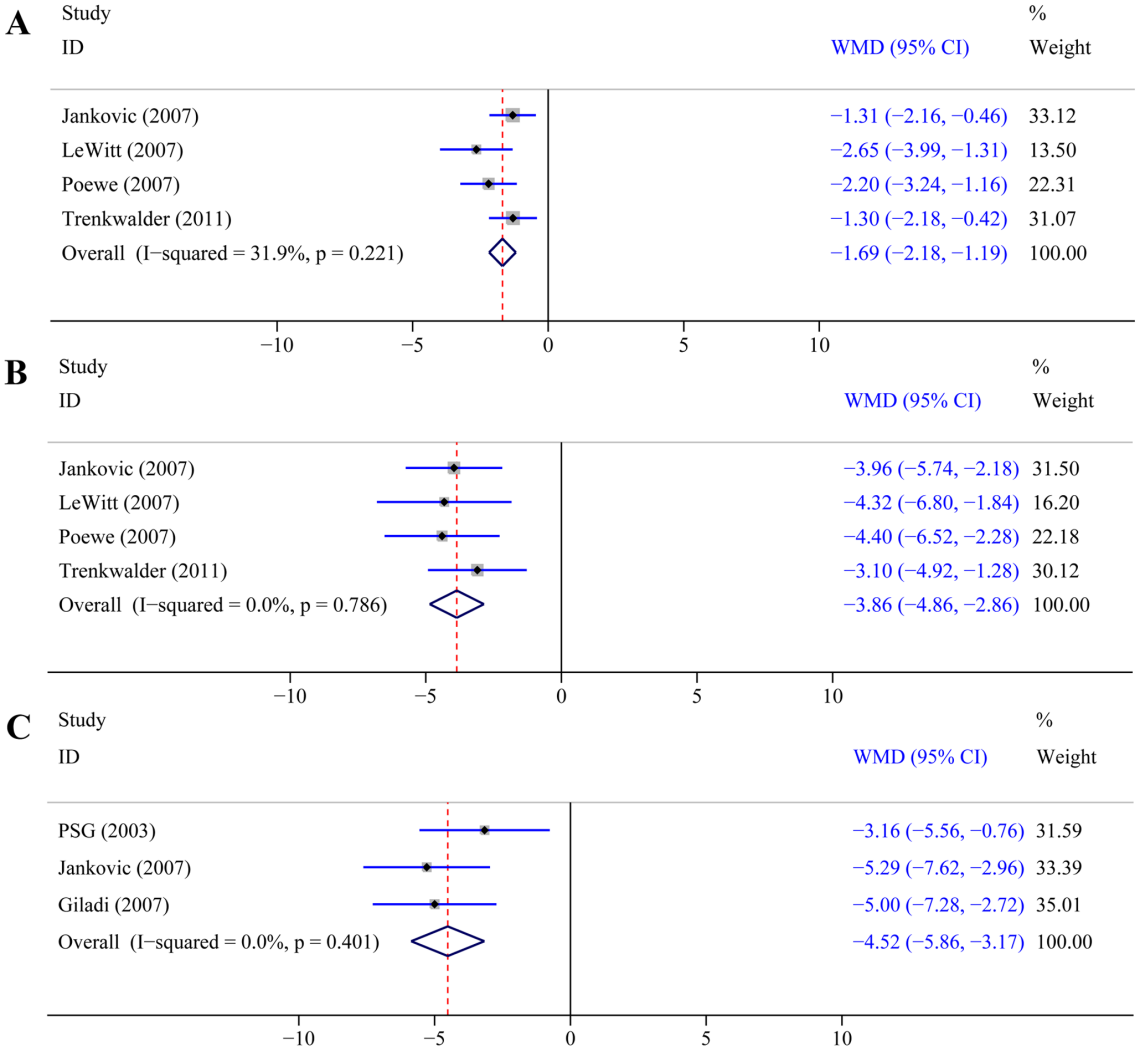
Impact of rotigotine versus placebo on UPDRS scores. Panel A: UPDRS ADL score. Panel B: UPDRS motor score. Panel C: UPDRS ADL and motor subtotal score. Abbreviations: UPDRS = unified Parkinson’s disease rating scale; ADL = activities of daily living.

All six trials evaluated the efficacy of rotigotine versus placebo using UPDRS scores (Table 2). In the four trials [20], [22]–[24] (n = 1105) that assessed UPDRS ADL score and motor score, patients had a greater response to rotigotine than placebo, as evidenced by significantly greater reductions from baseline in ADL score (WMD –1.69, 95% CI –2.18 to –1.19; P<0.0001; Figure 2A) and motor score (WMD –3.86, 95% CI –4.86 to –2.86; P<0.0001; Figure 2B). In the three trials [19]–[21] (n = 845) that assessed the UPDRS ADL and motor subtotal score in early PD, patients had a greater response to rotigotine than placebo, as evidenced by a significantly greater reduction from baseline in subtotal score (WMD –4.52, 95% CI –5.86 to –3.17; P<0.0001; Figure 2C).

**Table 2:** Efficacy and tolerability results of overall and subgroup analyses.

	Early and advanced PD		Early PD		Advanced PD+LD^a^	
Items	No. of trials	WMD/RR(95%CI)	No. of trials	WMD/RR(95%CI)	No. of trials	WMD/RR(95%CI)
UPDRS ADL score	4	–1.69 (–2.18, –1.19)	1	–1.31 (–2.16, –0.46)	2	–2.37 (–3.19, –1.55)
UPDRS motor score	4	–3.86 (–4.86, –2.86)	1	–3.96 (–5.74, –2.18)	2	–4.37 (–5.98, –2.75)
ADL+motor subtotal score	3	–4.52 (–5.86, –3.17)	3	–4.52 (–5.86, –3.17)	N/A	N/A
Overall withdrawals	6	0.88 (0.64, 1.21)	3	1.12 (0.84, 1.47)	2	0.69 (0.29, 1.61)
Withdrawals due to adverse events	6	1.82 (1.29, 2.59)	3	2.72 (1.57, 4.70)	2	1.40 (0.82, 2.37)

Abbreviations: UPDRS = unified Parkinson’s disease rating scale; PD = Parkinson’s disease; WMD = weighted mean difference; RR = relative risk; CI = confidence interval; LD = levodopa; ADL = activities of daily living; N/A = not available.

a +LD indicates that patients also took levodopa during the trials.

### Withdrawals

**Figure 3:**
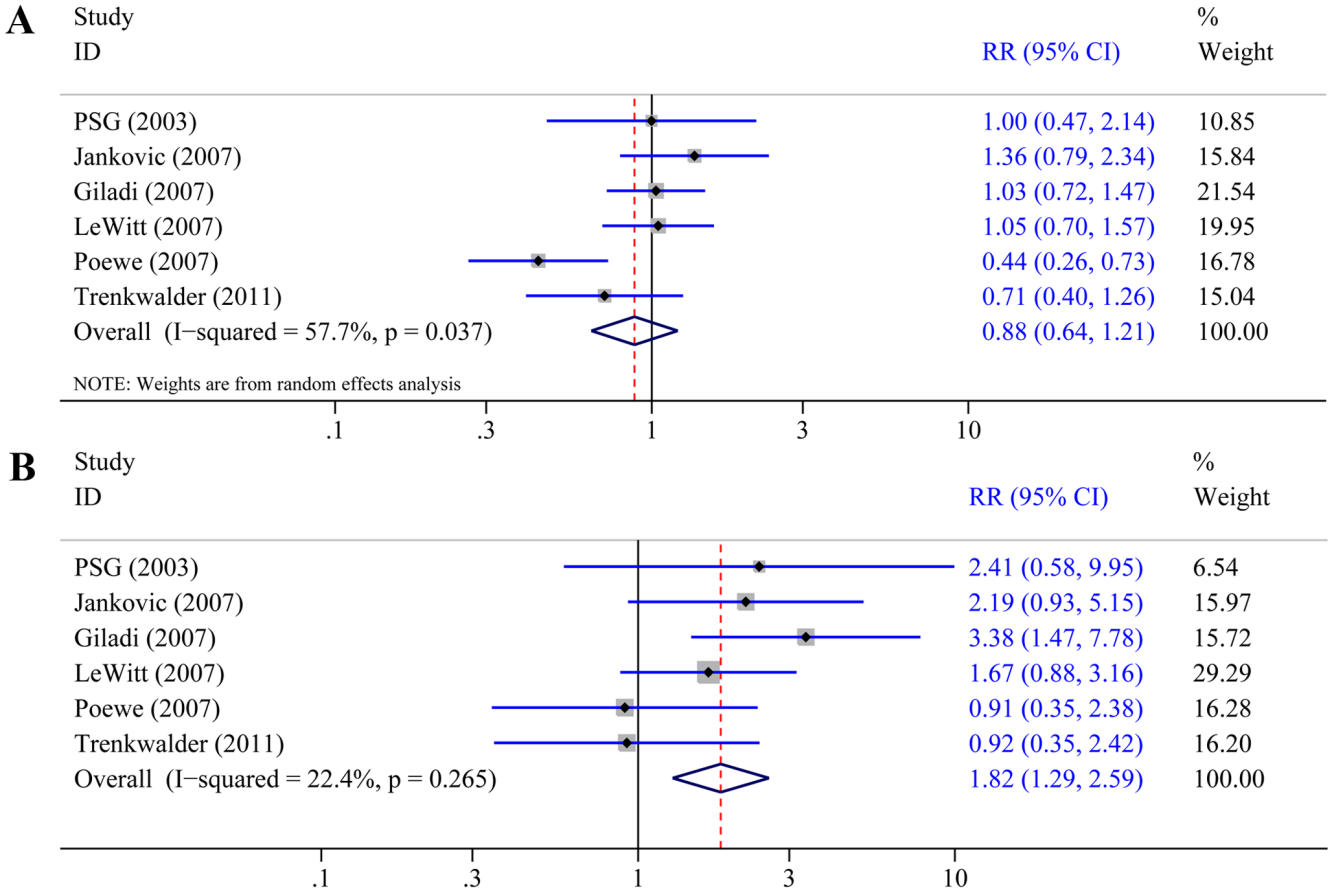
Effect of rotigotine versus placebo on withdrawals. Panel A: Overall withdrawals. Panel B: Withdrawals due to adverse events.

In all six trials [19]–[24] that reported the overall number of patient withdrawals, we found no difference between rotigotine and placebo (RR 0.88, 95% CI 0.64–1.21; P = 0.44; Figure 3A), with some evidence of heterogeneity between trials (P = 0.037, I^2^ = 57.7%). This heterogeneity was mostly explained by the atypical results of one study [23] in which there were significantly more withdrawals in the placebo group than in the rotigotine group (25.74% versus 11.27%). Patients withdrew from trial for various reasons. In the study by Poewe et al. [23], more patients withdrew due to unsatisfactory therapeutic effects and consent withdrawal in the placebo group than in the rotigotine group (14.85% versus 5.39%). This may be related to a slightly lower proportion of patients receiving concomitant antiparkinsonian drugs, in addition to levodopa, in the placebo group (43%) than in the rotigotine group (52%), and the double-dummy trial design (rotigotine, pramipexole, and placebo). Other factors that may contribute to the heterogeneity included the longer duration of PD in the trial by Poewe et al. [23] than in the other trials, and the mean daily dose of levodopa was higher in the trial by Poewe et al. than in another trial of advanced PD patients [22]. When we excluded the trial by Poewe et al. [23], there was no heterogeneity (P = 0.61, I^2^ = 0%) and no difference was also found between rotigotine and placebo (RR 1.03, 95% CI 0.83–1.27; P = 0.80). In all six trials that reported the number of patient withdrawals due to adverse events, the outcome occurred in 139/1215 (11.4%) patients treated with rotigotine, and in 37/577 (6.4%) patients treated with placebo. Rotigotine was associated with a significantly higher rate of withdrawals due to adverse events compared with placebo (RR 1.82, 95% CI 1.29–2.59; P = 0.0008; Figure 3B).

### Adverse Events

**Figure 4:**
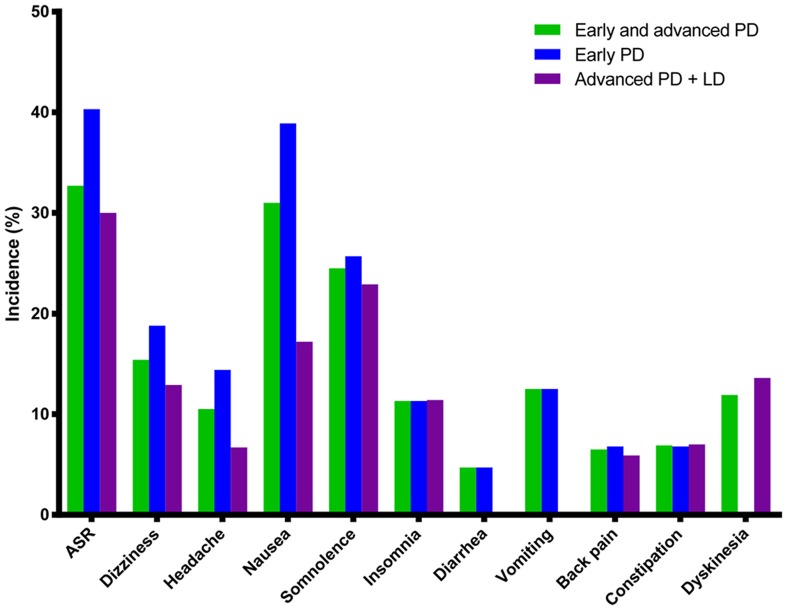
The pooled incidence of adverse events in PD patients treated with rotigotine. Abbreviations: ASR = Application site reactions; LD = levodopa.

All six trials reported adverse events with an incidence of ≥5% in the rotigotine group. The results of the meta-analysis of adverse events that were reported in at least three trials are summarized in Table 3 and the pooled incidence rates of adverse events in PD patients treated with rotigotine are presented in Figure 4. The most commonly reported adverse events and serious adverse events are discussed below.

**Table 3:** Safety results of overall and subgroup analyses.

	Early and advanced PD		Early PD		Advanced PD+LD^a^	
Adverse events	No. of trials	RR(95%CI)	No. of trials	RR(95%CI)	No. of trials	RR(95%CI)
Application site reactions	6	2.92 (2.29, 3.72)	3	3.01 (2.18, 4.16)	2	2.68 (1.81, 3.97)
Dizziness	6	1.47 (1.12, 1.95)	3	1.55 (1.05, 2.77)	2	1.32 (0.83, 2.08)
Headache	6	1.27 (0.92, 1.76)	3	1.42 (0.93, 2.17)	2	1.00 (0.55, 1.81)
Nausea	5	2.19 (1.70, 2.81)	3	2.32 (1.72, 3.14)	1	1.58 (0.84, 2.97)
Somnolence	5	1.42 (1.14, 1.77)	3	1.62 (0.93, 2.80)	2	1.25 (0.91, 1.71)
Insomnia	4	1.84 (1.16, 2.91)	3	1.79 (1.02, 3.13)	1	1.95 (0.87, 4.35)
Diarrhea	3	1.10 (0.56, 2.14)	3	1.10 (0.56, 2.14)	N/A	N/A
Vomiting	3	5.18 (2.25, 11.93)	3	5.18 (2.25, 11.93)	N/A	N/A
Back pain	3	1.21 (0.70, 2.10)	2	1.12 (0.59, 2.13)	1	1.49 (0.49, 4.49)
Constipation	3	1.43 (0.82, 2.50)	2	1.62 (0.77, 3.37)	1	1.20 (0.51, 2.83)
Dyskinesia	3	2.52 (1.47, 4.32)	N/A	N/A	2	2.75 (1.48, 5.13)

Abbreviations: PD = Parkinson’s disease; RR = relative risk; CI = confidence interval; ROT = rotigotine; PLA = placebo; LD = levodopa; vs = versus; N/A = not available.

a +LD indicates that patients also took levodopa during the trials.

**Figure 5:**
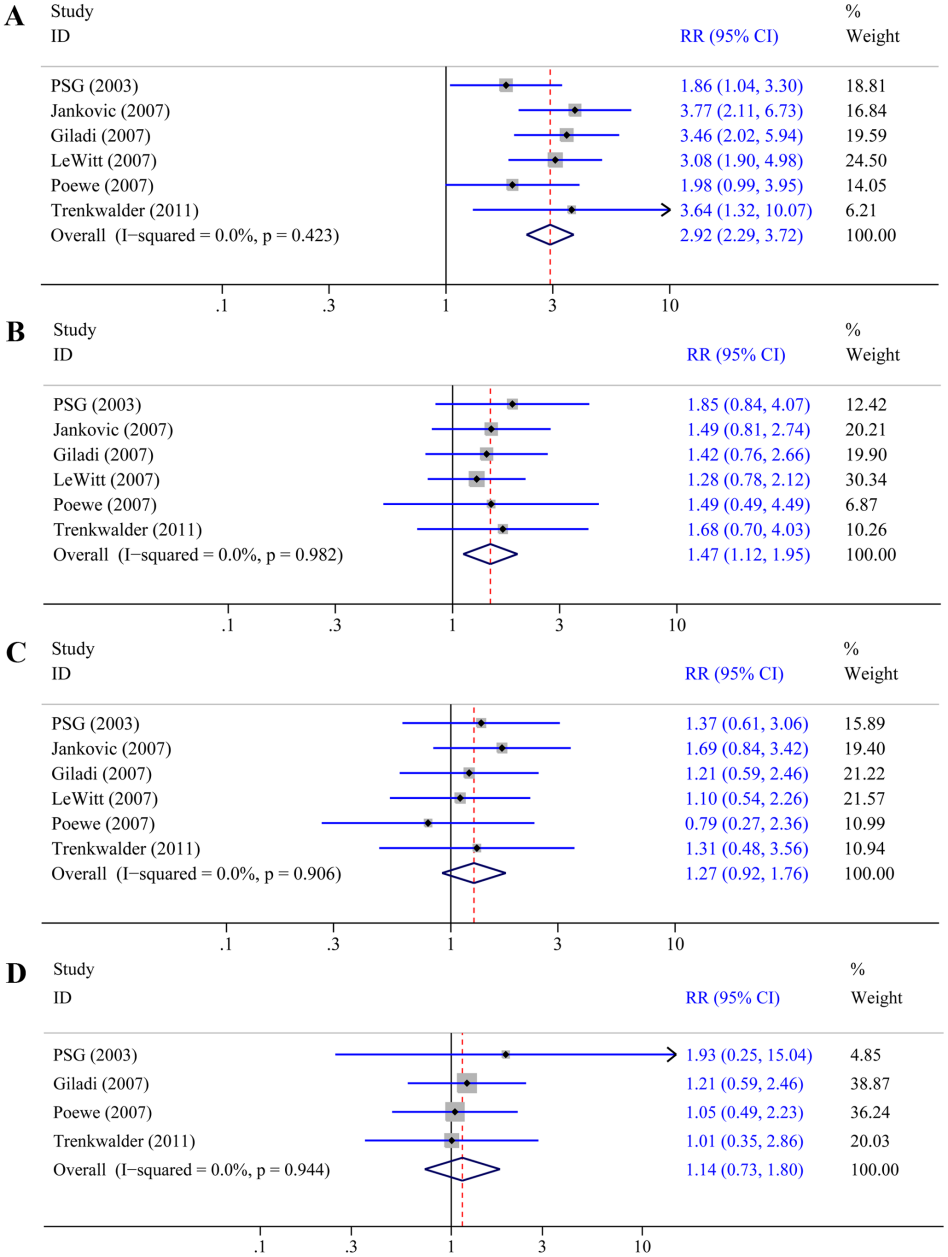
Effect of rotigotine versus placebo on the incidence of adverse events. Panel A: Application site reactions. Panel B: Dizziness. Panel C: Headache. Panel D: Serious adverse events.

All six trials reported the incidence of application site reactions and its incidence was significantly higher with rotigotine than with placebo (RR 2.92, 95% CI 2.29–3.72; P<0.0001; Figure 5A). The incidence of dizziness was reported in all six trials, and its incidence was significantly higher with rotigotine than with placebo (RR 1.47, 95% CI 1.12–1.95; P = 0.006; Figure 5B). In all six trails that reported the incidence of headache, no difference was found between rotigotine and placebo (RR 1.27, 95% CI 0.92–1.76; P = 0.15; Figure 5C). In the four trials [19], [21], [23], [24] that reported the incidence of serious adverse events, no difference was also found between rotigotine and placebo (RR 1.14, 95% CI 0.73–1.80; P = 0.56; Figure 5D).

### Subgroup and Sensitivity Analyses

In subgroup analyses, UPDRS scores and overall withdrawals were analyzed separately in early PD patients who had not taken levodopa and in advanced PD patients who had already taken levodopa (Table 2). The results of these subgroup analyses were consistent with the results for all PD patients combined. The number of patients who withdrew due to adverse events was significantly greater in the rotigotine group than in the placebo group among early PD patients (RR 2.72, 95% CI 1.57–4.70; P = 0.0004), but no difference was found in advanced PD patients (RR 1.40, 95% CI 0.82–2.37; P = 0.22). When the incidence of adverse events was reanalyzed in early PD patients who had not taken levodopa and in advanced PD patients who had already taken levodopa, the results were consistent with those reported for each of the meta-analyses in all patients, except for dizziness, nausea, somnolence, and insomnia (Table 3). Although rotigotine was associated with significantly higher incidences of dizziness, nausea, and insomnia in all patients and in early PD patients compared with placebo, no difference was found in advanced PD patients. Although rotigotine was associated with a significantly higher incidence of somnolence in all PD patients, no differences between rotigotine and placebo were found in early PD patients or in advanced PD patients.

In the analysis of overall withdrawals, there was statistically significant heterogeneity due to the study by Poewe et al. [23]. When this trial was excluded, no difference was also found between rotigotine and placebo (RR 1.03, 95% CI 0.83–1.27; P = 0.80). In the trial by Poewe et al. [23], application site reactions were reported as erythema and pruritus, but the exact incidence of application site reactions was not reported. Therefore, we used the total incidence of erythema and pruritus instead of the incidence of application site reactions and combined it with the incidences of application site reactions reported in the other five studies (RR 2.92, 95% CI 2.29–3.72; P<0.0001). After excluding the study by Poewe et al. [23], we reanalyzed the results to perform the sensitivity analysis. Finally, the results were consistent with those of the previous analysis (RR 3.07, 95% CI 2.37–3.99; P<0.0001). Since all the trials included had Jadad scores of 4 or higher and there were no open-label trails, we were unable to conduct these sensitivity analyses.

## Discussion

This is the first meta-analysis of randomized controlled trials to assess the efficacy, tolerability, and safety of rotigotine in PD patients. Our results demonstrated that rotigotine was associated with significant improvements in PD symptoms, as evidenced by reductions in UPDRS ADL score, motor score, and the ADL and motor subtotal score compared with placebo. The magnitude of reduction in UPDRS ADL score (–1.69) in the overall cohort of PD patients was slightly greater than that in early PD patients (–1.64) but smaller than that in advanced PD patients (–2.2) when we compared the results with those of two meta-analyses of dopamine agonists [25], [26]. In early PD patients who had not taken levodopa, the reduction in UPDRS ADL score (–1.31) was smaller than that reported for early PD patients (–1.64) in dopamine agonists [25]. In advanced PD patients who had already taken levodopa, the reduction in UPDRS ADL score (–2.37) was higher than that reported for advanced PD patients (–2.2) in the earlier study [26]. However, the reduction in UPDRS motor score (–3.86) was smaller than that reported for early PD patients (–5.32) and advanced PD patients (–5.56) [25], [26]. In the subgroup analysis, the effect sizes of rotigotine versus placebo in UPDRS motor score among early (–3.96) and advanced PD patients (–4.37) were small as compared with those of dopamine agonists in previous studies [25], [26]. This may be due to the shorter observation period of the rotigotine trials [19], [22]. In advanced PD, patients treated with rotigotine also exhibit a significant reduction in “off” time and an increase in “on” time without troublesome dyskinesia as compared with patients treated with placebo (data not shown). Although there were only two trials of advanced PD, the reduction in “off” time (–1.56) was similar to that reported for dopamine agonists (–1.2) [26]. In the present study, the reductions in UPDRS motor score and “off” time were statistically significant and meet the newly suggested criteria for clinical relevance where reductions in motor score of 3.5 points and “off” time of 1 hour are considered to be the minimal clinically important changes in early and advanced PD [27].

Although rotigotine was not associated with a significant increase in the overall withdrawals from trials, it was associated with a significant increase in withdrawals due to adverse events (11.4%) compared with placebo (6.4%). The rate of withdrawals due to adverse events among rotigotine-treated patients was similar to that in meta-analyses of dopamine agonists (11.9%) and monoamine oxidase type B inhibitors (10.2%) [25], [28]. In our subgroup analysis, rotigotine was associated with a significant increase in the rate of withdrawals due to adverse events in early PD patients but not in advanced PD patients as compared with placebo. The results in advanced PD patients were not statistically significant and could be attributed to the finding that one trial included worsening of PD as an adverse event [22].

The results of pooled incidences of adverse events showed that application site reactions, nausea, and somnolence were the most common adverse events in the overall cohort of patients and in subgroups of early and advanced PD patients who received rotigotine. These findings were consistent with those of a study of rotigotine in early and advanced PD patients aged <65 or ≥65 years [29]. Although a study of ropinirole previously evaluated some of the adverse events associated with rotigotine [30], the fundamental objective was to evaluate the safety of ropinirole; therefore, application site reactions and serious adverse events, in particular, were not assessed. In the present study, more trials were included and adverse events reported in at least three trials were studied. We found that application site reactions were the most common adverse events and patients treated with rotigotine had about threefold greater risk of developing application site reactions in the overall cohort and in subgroups of early and advanced PD patients. Erythema and pruritus were the most commonly reported skin reactions. Although application site reactions occurred in up to 32.7% of patients in the rotigotine group, most events were reported to be mild to moderate, and the incidence was similar to that of other transdermal patches, including rivastigmine (31%) [31]. However, in the subgroup analysis, the incidence of application site reactions was higher in early PD patients than in advanced PD patients. This was probably due to one study [23] that reported the incidences of erythema and pruritus, but not other skin reactions.

In the present study, we evaluated four gastrointestinal side effects including nausea, diarrhea, vomiting, and constipation. Although rotigotine is delivered through a transdermal patch, early PD patients had about fivefold greater risk of developing vomiting and about twofold greater risk of developing nausea when treated with rotigotine compared with placebo. These results are consistent with those reported by Kulisevsky et al. [30]. However, rotigotine was not associated with increased risks of diarrhea and constipation. Rotigotine also increased the risk of dyskinesia by about twofold in advanced PD patients. In the overall cohort of PD patients and early PD patients, rotigotine was also associated with significantly increased risks of dizziness and insomnia but not of serious adverse events, headache, or back pain. In the overall and subgroup analyses, we found some discrepancies in the incidences of nausea, somnolence, dizziness, and insomnia. Because of the small number of studies available for the subgroup analyses, further studies are needed to clarify the impact of rotigotine on the incidence of these events.

There are some limitations that should be mentioned to appropriately interpret the results of our study. First, in some comparisons, especially in subgroup analyses, there were only one or two clinical trials available, sometimes with a relatively low incidence of events, which means that the results of these analyses cannot be generalized. Second, the safety and tolerability variables were considered as secondary objectives, and most trials did not report adverse events with incidences of <5%. Furthermore, a classic bias in analyses of adverse events in randomized controlled trials is the inclusion of studies with short observation periods. Therefore, the conclusions can only be drawn for the most common adverse events and the overall incidence of adverse events might have been underestimated. Finally, the assessment of publication bias was not performed because only six trials were included in the present study.

In conclusion, our meta-analysis showed that the use of rotigotine transdermal patch can reduce the symptoms of PD. However, rotigotine was also associated with a higher incidence of adverse events, especially application site reactions, compared with placebo. Although refrigerated storage of rotigotine transdermal patch at 2–8°C can reduce the development of crystals, the definitive resolution of the problem is to reformulate the drug product. The manufacturer recently reformulated the patch [32], and the reformulated patch can be stored at room temperature without the development of crystals. Further studies are also necessary to evaluate the reformulated patch.

## Supporting Information

File S1 **PRISMA 2009 Checklist for the meta-analysis.**
(DOC)
